# Optimizing qualitative methods in implementation research: a resource for editors, reviewers, authors, and researchers to dispel ten common misperceptions about qualitative research methods

**DOI:** 10.1186/s13012-025-01474-z

**Published:** 2025-12-04

**Authors:** Andrea L. Nevedal, Christine P. Kowalski, Erin P. Finley, Gemmae M. Fix, Alison B. Hamilton, Christopher J. Koenig

**Affiliations:** 1 VA Center for Clinical Management Research (CCMR), VA Ann Arbor Healthcare System, 2215 Fuller Rd, Mail Stop 152, Ann Arbor, MI 48105 USA; 2 VA Health Systems Research Center for the Study of Healthcare Innovation, Implementation, and Policy, VA Greater Los Angeles Healthcare System, Los Angeles, CA USA; 3 Departments of Medicine and Psychiatry and Behavioral Sciences, Long School of Medicine, UT Health San Antonio, San Antonio, TX USA; 4 Center for Health Optimization and Implementation Research (CHOIR), VA Bedford and Boston Healthcare Systems, Bedford/Boston, MA USA; 5 Section of General Internal Medicine, Boston University Chobanian and Avedisian School of Medicine, Boston, MA USA; 6 Department of Psychiatry and Biobehavioral Sciences, David Geffen School of Medicine, University of California Los Angeles, Los Angeles, CA USA; 7 Communication Studies College of Liberal and Creative Arts, San Francisco State University, San Francisco, CA USA; 8 Medical Cultures Lab, University of California, San Francisco, San Francisco, CA USA

**Keywords:** Qualitative methods, Rigor, Mixed methods, Implementation science, Evaluation standards, Subjectivity, Qualitative analysis

## Abstract

**Background:**

Qualitative methods are central to implementation research. Qualitative research provides rich contextual insight into lived experiences of health and illness, healthcare systems and care delivery, and complex implementation processes. However, quantitative methods have historically been favored by editors and reviewers who serve as gatekeepers to scientific knowledge. Thus, we underscore that editors and reviewers must be familiar with the underlying principles and strengths of qualitative methods to avoid perpetuating inappropriate evaluation criteria that hinder qualitative research dissemination and funding opportunities. We aim to help authors and researchers provide sufficient details to dispel misperceptions and editors and reviewers to better evaluate studies using qualitative methods to maximize dissemination for high-impact implementation research.

**Methods:**

We convened a panel of six researchers with extensive experience in: designing, conducting, and reporting on qualitative research in implementation science and other healthcare research; training and mentoring others on qualitative methods; and serving as journal editors and manuscript/grant peer reviewers. We reviewed existing literature, published and unpublished reviewer critiques of qualitative grants and manuscripts, and discussed challenges facing qualitative methodologists when disseminating findings. Over the course of one year, we identified candidate topics, ranked each by priority, and used a consensus-based process to finalize the inventory and develop written guidance for handling each topic.

**Results:**

We identified and dispelled 10 common misperceptions that limit the impact of qualitative methods in implementation research. Five misperceptions were associated with the application of inappropriate quantitative evaluation standards (subjectivity, sampling, generalizability, numbers/statistics, interrater reliability). Five misperceptions were associated with overly prescribed qualitative evaluation standards (saturation, member checking, coding, themes, qualitative data analysis software). For each misperception, we provide guidance on key considerations, responses to common critiques, and citations to appropriate literature.

**Conclusions:**

Unaddressed misperceptions can impede the contributions of qualitative methods in implementation research. We offer a resource for editors, reviewers, authors, and researchers to clarify misunderstandings and promote more nuanced and appropriate evaluation of qualitative methods in manuscripts and grant proposals. This article encourages a balanced assessment of the strengths of qualitative methods to enhance understandings of key problems in implementation research, and, ultimately, to strengthen the impact of qualitative findings.

**Supplementary Information:**

The online version contains supplementary material available at 10.1186/s13012-025-01474-z.

Contributions to the Literature

- Although qualitative research methods are integral to implementation science and health services research, qualitative researchers have historically encountered barriers to disseminating high-impact results.
- This article identifies 10 misperceptions that impede appropriate review of qualitative research. Five misperceptions involve inappropriately applying quantitative research standards to qualitative research (subjectivity, sampling, generalizability, numbers/statistics, interrater reliability) and five misperceptions include overly prescribing standards about qualitative research methods (saturation, member checking, coding, themes, software).
- We offer guidance to qualitative researchers to preemptively address misperceptions about qualitative methods.
- We offer guidance to journal editors and reviewers seeking to ensure appropriate and impartial assessment of qualitative research.

## Background

**Fig. 1:**
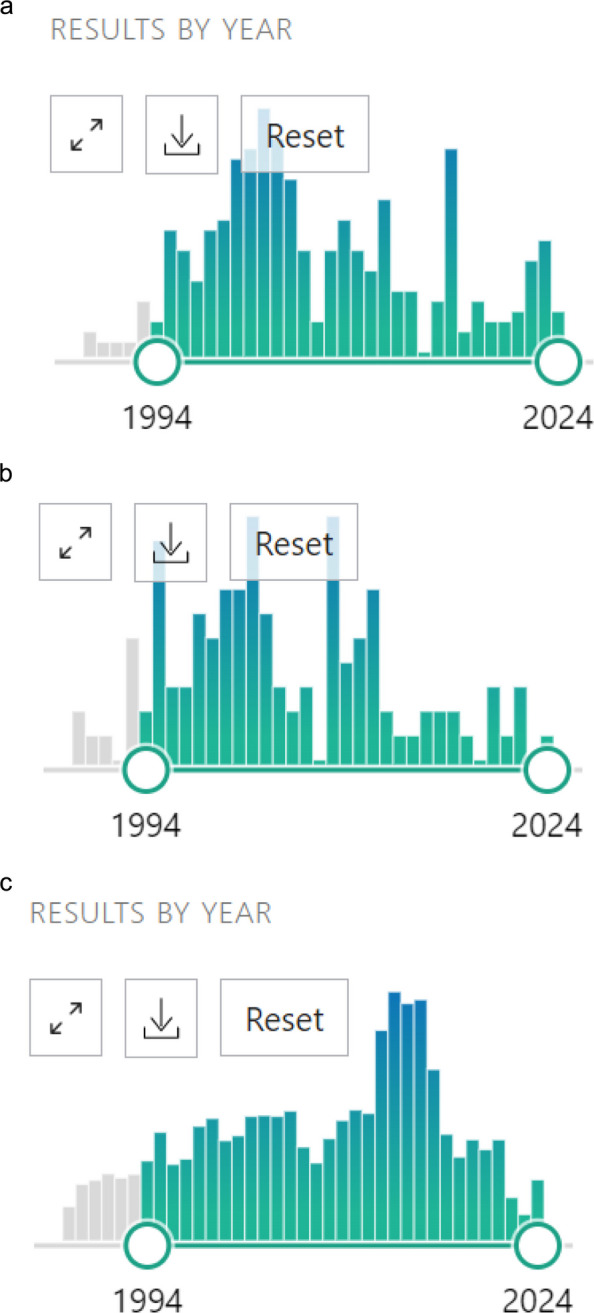
**a** PubMed Search Results: (Qualitative research) AND (“BMJ (Clinical research ed.)”[Journal]) = 303 articles. **b** PubMed Search Results: (Qualitative methods) AND (“BMJ (Clinical research ed.)”[Journal]) = 111 articles. **c** PubMed Search Results: (Statistics) AND (“BMJ (Clinical research ed.)”[Journal]) = 7,066 articles

In implementation science and other related disciplines such as health services research, researchers want to fund and disseminate their work but may experience frustrations during the peer-review process. In addition to common challenges of writing high-quality grants and manuscripts, qualitative researchers face unique constraints. Historically, qualitative research has faced greater barriers to funding and publication because scientific norms have privileged quantitative, statistically driven methods over qualitative approaches that examine human experience and social processes [1–15]. As a result, qualitative researchers have fewer opportunities for securing funding and publishing in high-impact journals and this is problematic because their insights are not included as extensively in the broader scientific discourse (See Fig. 1a-c) [1–12, 14, 16]. Therefore, qualitative researchers need to identify funding sources and peer-reviewed journals amenable to qualitative research. Program officers and editors must find skilled reviewers with expertise in providing impartial and constructive reviews, who are knowledgeable enough about diverse qualitative research to appropriately evaluate methods, findings, and impact [15].

Over the past two decades, qualitative and mixed methods research have grown in prominence in implementation science [17–22]. The Qualitative Research in Implementation Science (QualRIS) expert working group convened by the National Cancer Institute identified seven essential ways qualitative methods are used in implementation science, including to elicit participant or partner-centered perspectives, inform design and implementation, understand context across diverse settings, provide documentation and encourage reflection, gain insight into implementation effectiveness, understand mechanisms of change, and contribute to theoretical development [19]. Despite diverse ways in which qualitative methods can enhance quality and impact of implementation research and practice, qualitative researchers still encounter editors and reviewers who have insufficient training and knowledge of the full range of qualitative methods and epistemologies to appropriately evaluate qualitative study designs [1–13, 15]. The QualRIS report also noted, “many implementation scientists have limited or no experience in qualitative methods” [19]. More recent literature highlights a need for improving implementation researchers’ competency in qualitative research methods [23–25]. For example, the updated Consolidated Framework for Implementation Research (CFIR) user guide highlights common questions about qualitative methods [26]. Consequently, when reviewers trained primarily in quantitative traditions assess qualitative research, they may apply inappropriate evaluative criteria, producing critiques that reflect entrenched disciplinary biases and misperceptions regarding the aims and standards of qualitative inquiry [13, 27]. In response, qualitative researchers may feel pressured to incorporate language that aligns with these inappropriate standards to make their work more acceptable during peer-review [13, 28]. For example, one of this manuscript’s authors received the following rejection explanation from an editor-in-chief of a prominent journal:

*We are sorry to report we cannot accept your manuscript for publication. Your paper provides an interesting first-person narrative of [clinician] views on [topic]. Our concern was the small sample size, potential problems with representativeness of the sample relative to [medical specialty] practice, and thus the generalizability of the conclusions. We unfortunately have had to lower the priority for this paper relative to others currently being considered.*

This explanation typifies fundamental misunderstandings related to several aspects of qualitative research, including sample size, representativeness, and generalizability of findings. Inappropriate reviews and editorial rejections further reinforce the privileging of quantitative over qualitative research [27]. Biased review and editorial practices sustain the dominance of quantitative paradigms, rewarding qualitative studies that mimic quantitative norms and marginalizing those that exemplify qualitative rigor on its own terms [27].

Given the importance of qualitative methods in advancing implementation science and practice, the objective of this article is to identify and dispel misperceptions that hinder advancement and dissemination of qualitative research. This article is intended to be a practical resource for two audiences within and outside of implementation science: 1) authors and researchers responding to inappropriate critiques of qualitative methods, and 2) editors and reviewers seeking to better understand and impartially evaluate qualitative methods.

## Methods

We convened six researchers with extensive experience in designing, implementing, and reporting on qualitative research in implementation science and other healthcare research; training and mentoring others on qualitative methods; and serving as journal editors and manuscript/grant peer-reviewers. We have diverse educational backgrounds in anthropology, linguistics and communication, implementation science, health services research, and epidemiology. All authors routinely conduct implementation research in a range of settings.

Over the course of 12 months via virtual meetings and emails, we used a process informed by Critical Reflective Inquiry to investigate the challenges of advancing qualitative research [29–32] to offer guidance to editors, reviewers, authors, and researchers. First, we generated a list of topics. We then used an iterative process of reviewing existing literature, published and unpublished reviewer critiques of qualitative grants and manuscripts, and challenges facing implementation scientists. The first and senior authors met initially to reflect on common misperceptions of qualitative research and proposed five initial key topics. Then, all authors reviewed the initial five topics and through group discussions proposed nine additional candidate topics based upon common challenges encountered when disseminating implementation science findings that rely on qualitative methods.

Second, we prioritized and categorized the topics. We listed the 14 topics in a shared document. Using a consensus-based process to discuss differences in rankings, we collaboratively ranked each topic based on higher and lower priority for inclusion. We reduced the list to 10 topics with the highest priority by consensus. We then reorganized the 10 topics into two groups of misperceptions about qualitative research. Misperceptions 1–5 address inappropriate use of quantitative standards to assess qualitative methods. Misperceptions 6–10 address inappropriately rigid expectations for qualitative methods.

Third, we developed written guidance related to each misperception. We assigned a primary and secondary author to write the initial draft for each misperception and then all authors reviewed guidance for each misperception. The resulting guidance for each misperception includes: 1) a description of the problem and why the critique may be inappropriate; 2) references to related literature; 3) special considerations for implementation science; 4) suggestions for reviewers to provide more informed critiques; and 5) strategies for authors to anticipate and address inappropriate reviewer critiques by preparing more rigorous methods descriptions.

## Results

**Fig. 2:**
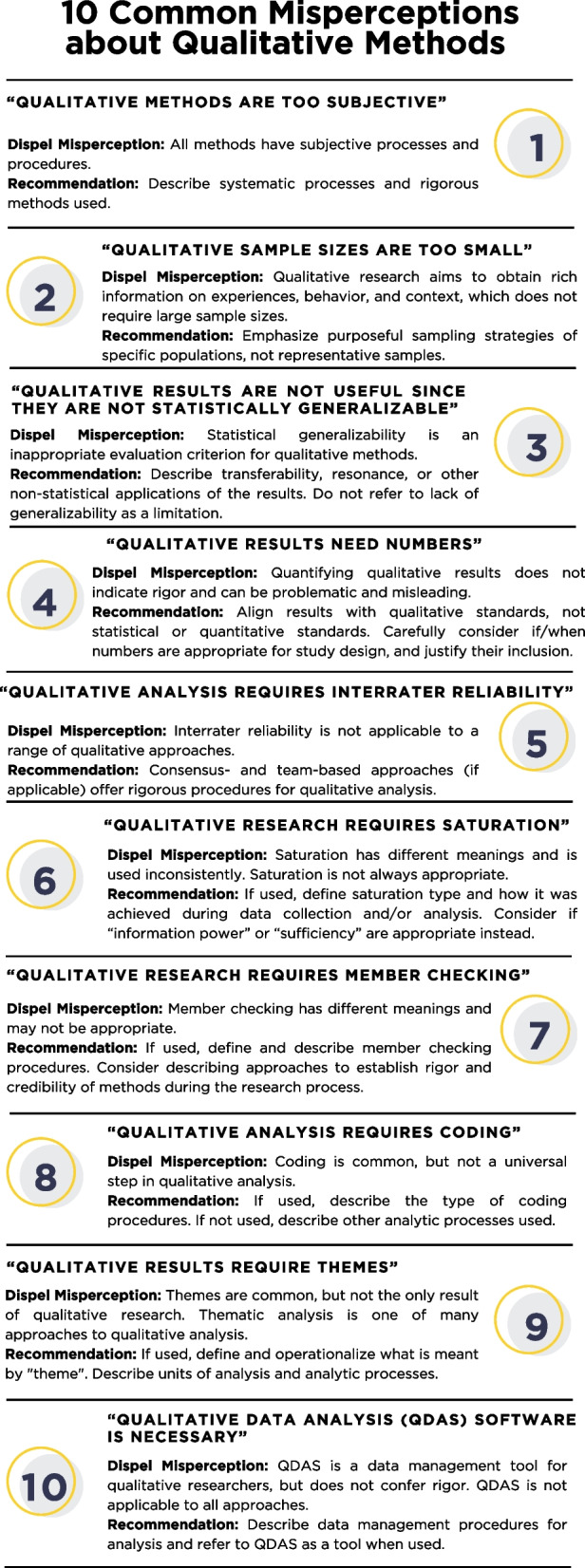
10 Common Misperceptions about Qualitative Methods

The following sections provide guidance on how to dispel 10 misperceptions regarding qualitative methods, including specific considerations for implementation science. Tables 1 and 2 provide considerations for both authors aiming to dispel misperceptions and reviewers to impartially evaluate qualitative methods. Figure 2 provides an infographic about the 10 misperceptions. Additional File 1 provides questions for editors and reviewers to assess their own misperceptions about qualitative methods.

**Table 1:** Considerations for authors to dispel misperceptions 1–5 and for reviewers to more appropriately evaluate qualitative methods

Misperception	Example Critique	Dispel Misperception	Author Considerations	Reviewer Evaluation Considerations
(1) “Qualitative methods are too subjective.”	“The utility of qualitative methods is questionable…”	• All methods have subjective processes and procedures • Qualitative research serves a different purpose and utilizes different data than quantitative methods and has autonomy as a method	• Describe systematic processes and rigorous methods used, including the purpose and the data being analyzed • Consider commenting on reflexivity (e.g., background, training, positionality, power dynamics)	• What methodological approach and analytic procedures do the authors describe? (e.g., rapid analysis, content analysis, grounded theory, ethnography, discourse analysis) • Do authors reference literature for methods? • How do the authors describe their credentials and training in qualitative methods? Is there a qualitative expert overseeing the work?
(2) “Qualitative sample sizes are too small.”	“Our concern is the small sample size of 26…”	• Qualitative research seeks to obtain rich information on experiences, behaviors, and contexts, which does not require large sample sizes • Purposeful sampling varies by design, population, social context, data types, and analytic methods	• Emphasize and describe purposeful sampling strategies of specific populations, and explain why the approach was adopted	• Do the authors identify and define the type of purposeful sampling used in the study? • Do the authors discuss sampling in relation to study aim(s), research question/s, and design? • What rationale is provided for their sampling approach/size? Where appropriate, do authors cite relevant literature for the approach/size?
(3) “Qualitative results are not useful since they are not statistically generalizable.”	“Our concern is the lack of generalizability of conclusions…”	• Qualitative research is not meant to be statistically generalizable. Rather, it is intended to provide detailed information regarding a specific group, setting, or problem • Qualitative researchers provide rich description so that the reader can determine if the results are transferable or resonate	• Describe transferability of results (e.g., applicability, resonance, theoretical engagement, “conceptual generalizability”) •Do not refer to lack of generalizability as a limitation	• How do the authors describe the participants and research setting? • Do the authors note how results may be applicable to other problems/contexts? • Do authors present methods and results so they are understandable and/or resonate to the reader? • Do authors state if theories, models, frameworks, or literature are used to support transferability?
(4) “Qualitative results need numbers.”	“The results can be improved with inferential statistics…”	Quantifying or adding numbers to qualitative results does not legitimize the results nor does it add rigor and can, in fact, be problematic and misleading	• Emphasize how results were in accordance with recommendations for rigorous qualitative research and research goals	• How do authors describe identifying patterns and the salience of results to participants? • If numbers/statistics are used, do authors provide rationale for including numbers/statistics and does that improve the clarity/salience of results?
(5) “Qualitative analysis requires inter-rater reliability (IRR).”	“The authors do not report inter-rater reliability of coding…”	• Qualitative research encompasses various approaches that are unsuitable for IRR (e.g., single analyst, semi-structured methods) • IRR emphasizes coding reliability over validity, which is problematic • IRR is not required in qualitative coding and, depending on the research question, may be misleading or less rigorous than other approaches, such as consensus-based coding	• Emphasize that (if applicable) consensus and team-based approaches are often more appropriate because they support dialogue, reflexivity, and integration of diverse perspectives in qualitative analysis	• How do authors describe (collaborative or individual) analytic processes, such as coding (see misperception 8)? • Do authors cite relevant literature for established processes and procedures for analysis? • If the authors use IRR, does this approach align with the qualitative method used? • What rationale and relevant literature do authors use to justify their approach?

**Table 2:** Considerations for authors to dispel misperceptions 6–10 and for reviewers to more appropriately evaluate qualitative methods

Misperception	Example Critique	Dispel Misperception	Author Considerations	Reviewer Evaluation Considerations
(6) “Qualitative research requires saturation.”	“Saturation is not mentioned…”	• Saturation is not appropriate for all qualitative studies and sampling approaches. Saturation is also debated in qualitative research because it may not fit a study’s aim or research design • Saturation has different meanings and is used inconsistently	• If used, define saturation type and where and how it was used in the relevant work • Consider “information power” or “sufficiency” if more appropriate to the research question or context	• If authors discuss saturation, how is saturation defined and operationalized for the study (e.g., thematic saturation)? • If saturation is not mentioned, do the authors describe other approaches for assessing data sufficiency, such as “information power”? • Is there a rationale and citation for the approach used in the study?
(7) “Qualitative research requires member checking.”	“I found no mention of member checking to document validity…”	• Member checking procedures are not appropriate for all studies and have been critiqued • Member checking is only one technique qualitative researchers use to establish credibility of findings	• If used, define, and describe member checking procedures • Consider describing ways of establishing rigor and credibility in the qualitative methods being reflexively performed during the research process	• If authors refer to member checking, how is it defined and when, how, and with whom does it occur? How are the “results” of the member checking incorporated? • What (other) techniques do the research use to establish credibility (e.g., considering how findings compare/contrast with prior research, describing steps taken to ensure participants feel comfortable in sharing sensitive information, describing steps taken in analysis to identify patterns and mitigate potential bias)? • What are ways that verification and validation strategies are incorporated during the research process rather than post-hoc?
(8) “Qualitative analysis requires coding.”	“There is no mention of codes…”	Coding is a common, but not universal, procedure for identifying meaningful segments in qualitative data for analysis	• If used, describe coding procedures • If not used, describe the analytic process employed	• If coding is discussed, do authors provide a rationale for the coding-based approach to address their research question(s)? What were the roles/processes used in coding data? Is there clear and logical description of how codes were developed and refined into a final codebook? • If coding is not discussed, what analytic procedures did the authors use? Do they provide a clear rationale and cite literature to support their approach?
(9) “Qualitative results require themes.”	“What are the themes?”	Themes are a common, but not universal, result of qualitative analysis	• If used, define, and operationalize what is meant by a ‘theme.’ • If not used, describe units of analysis and analytic approach	• How are results reported? Do results align with the research question(s) and analytic approach described? • If themes are generated, how do authors define “theme”? Do authors cite literature to support their thematic approach? • If themes are not generated, are results presented in a manner that is cogent and credible given study methods? • Do authors specify and cite literature to support their approach?
(10) “Qualitative Data Analysis Software (QDAS) is necessary.”	“Authors do not mention use of software for data analysis…”	QDAS can assist in qualitative data management and analysis but is not essential and is not necessary for all research projects and/or teams	• Describe rationale and procedures for data management and analysis, which may or may not include QDAS • Do not refer to QDAS as a proxy for what was done analytically	• If QDAS is used, what is the name and version? Do authors provide adequate description of the tasks for which they used QDAS? Do the authors describe next steps in analysis after QDAS is used? • If QDAS is not used, are data management procedures or processes described clearly? • Are procedures appropriate to the dataset (e.g., size, complexity) and research question(s)?

### Misperceptions 1–5: applying inappropriate quantitative evaluation standards to qualitative methods

#### Misperception 1: “Qualitative methods are too subjective”

One critique frequently levied against qualitative research is the misperception that qualitative methods are “too subjective” to produce high quality evidence. Prior studies have shown that even when using quantitative methods, which is construed as more “objective,” researchers who analyzed the same quantitative dataset had different results, indicating that subjectivity is inherent to analysis [33]. Thus, researchers have argued that all scientific methods have inherently subjective processes and procedures [12, 34].

The subjectivity misperception raises questions about what constitutes subjectivity. Whereas quantitative researchers aim to tightly control subjectivity, qualitative researchers adopt more nuanced perspectives that range from minimizing to celebrating the subjective for different reasons [34, 35]. Qualitative methods address different purposes, answer different questions, and analyze different data types than quantitative methods [12].

In response to the critique of qualitative findings as “too subjective,” qualitative researchers can address concerns about perceived subjectivity by linking their study aims and research questions to the rationale for why qualitative methods are used. For example, common qualitative questions in implementation science involve understanding staff perspectives regarding the uptake of an evidence-based innovation. Researchers should describe systematic methods they use by referencing appropriate standards that align with their approach and/or reflexive processes used to conduct high-quality qualitative research [15, 28, 34, 36–46]. Given implementation scientists have been predominately trained in quantitative methods [19], it is crucial that these editors, reviewers, and authors are mindful of how qualitative and quantitative methods have different goals, approaches, rigorous standards, and evidence, so that erroneous objectivity and subjectivity debates are not perpetuated.

#### Misperception 2: “Qualitative sample sizes are too small”

One common reason journal editors do not send qualitative manuscripts for peer-review (i.e., desk reject) is the perception that qualitative studies employ a “sample too small” to justify the findings. Whereas quantitative study designs can calculate an appropriate sample using statistical techniques, such as a power calculation, to test hypotheses, qualitative research sample size must be considered in light of the research question(s), overall study design, population, social context, data types, and analytic methods [47–52]. Further, what counts as a sufficient sample size in qualitative research varies widely (e.g., from 10 to 70 people) [30]. Qualitative methods aim to produce deep, contextual insights into experiences, in contrast to the generalized patterns derived from quantitative metrics like averages or statistical distributions.

The small sample size critique is a misapplication of quantitative research standards imposed on qualitative research. High-quality sampling can be best evaluated when authors both name their specific sampling method and justify its selection in relation to the study’s goals. Qualitative methods traditionally use either theoretical or purposeful sampling strategies, defined as nonprobability sampling to generate rich information, diverse participants, or rich cases [53]. Specific techniques for purposeful sampling include maximum variation, homogeneity, critical or typical case, criterion, opportunistic, and snowball techniques [53]. Each sampling type is used for particular reasons. For example, while homogenous sampling needs fewer cases, heterogenous and maximum variation sampling need more cases [47–49, 54].

Second, sample sizes alone should not be an indicator of the quality of reported results. Smaller sample sizes that are “information-rich” rely on accessing specific or diverse knowledge or experience [44]. As a result, data collection seeks to understand participants’ experiences and local contexts and may not require large sample sizes. In implementation science, a typical approach is to develop a sample of key informants who have specific knowledge relevant to research aims, such as knowledge of the population that is the target of the evidence-based practice. These samples may be inherently small at a given site, but their knowledge is critical for progressing with implementation [44]. In summary, editors and reviewers should avoid hasty assumptions about small sample sizes and assess sample size (and its justification) in relation to study aims and methods.

#### Misperception 3: “Qualitative results are not useful since they are not statistically generalizable”

A common critique from reviewers is that qualitative research is not impactful since it is not “generalizable,” which typically means *statistical* generalizability [2, 55, 56]. Statistical generalizability means that results are representative of and applicable to the broader population or question of interest [55]. Statistical generalizability does not apply to qualitative methods because “random representational samples are not used” [38]. Instead, the goal of qualitative research is to obtain “a rich, contextualized understanding of some aspect of human experience through the intensive study of particular cases” [55]. Qualitative research provides in-depth contextual details and a holistic picture about a particular setting, a population, or phenomena that cannot be obtained through quantitative research.

Rather than applying erroneous standards of statistical generalizability, *transferability* is more appropriate for assessing the merit of qualitative research. Transferability is the extent to which the reader determines if qualitative results can be applied to their own circumstances and/or other contexts, settings, or participants [36, 38, 42, 57–61]. Transferability has also been discussed as “resonance” or “conceptual generalizability” [42, 60]. Qualitative researchers can enhance the transferability of their findings in multiple ways [42, 61]. *Transferability as applicability* is when qualitative researchers provide rich description and nuance and, if appropriate to the study design, include firsthand accounts of participants’ perspectives (e.g., about implementation processes), so readers can evaluate how results relate to other contexts [61]. *Transferability as resonance* is when qualitative researchers share results so they are understandable, familiar, and recognizable to the reader [42, 61]. *Transferability as theoretical engagement* is when qualitative researchers use theories, models or frameworks (TMFs) to explain their results to the reader [61]. TMFs are important in implementation science because they provide a common language for researchers to explain results and for readers to interpret results across different contexts or innovations [62–64]. For example, the CFIR [65] and the Reach, Effectiveness, Adoption, Implementation, and Maintenance (RE-AIM) framework [66] are commonly used implementation science frameworks that help qualitative researchers demonstrate the transferability of findings by illuminating implementation determinants and outcomes. Additionally, qualitative researchers can help readers evaluate transferability by making explicit how findings may be transferable to other circumstances or social contexts [38, 55, 57–60].

Overall, reviewers and editors must recognize that statistical generalizability should not be used to assess the value of qualitative results. Generalizability is not a goal of qualitative research, and therefore not an appropriate standard nor a “limitation” [2, 55]. Instead, we encourage qualitative researchers to articulate how findings may be transferable for readers to determine which the results might be applicable to other contexts [42, 61].

#### Misperception 4: “Qualitative results need numbers”

The use of numbers in qualitative methods has long been controversial [59, 67]. Reviewers may assume that incorporating numbers (or “counts”) will legitimize qualitative findings. However, qualitative researchers have debated the use of numbers, resulting in general consensus that numbers and statistical analyses do not inherently enhance qualitative results and that there are important considerations when enumerating qualitative data [27, 67–69]. For example, the goal of qualitative methods in implementation science is often to gain an in-depth understanding, such as how and why people use innovations, in addition to how much and how often an innovation is used [14, 18, 19, 70, 71].

Enumerating qualitative data is often problematic. Counting can frame qualitative findings in ways that reinforce quantitative evaluation standards and can reduce the significance of key findings or evidence to a number [68]. Counting can also mislead interpretation of qualitative results (e.g., low frequencies overshadow important insights from smaller datasets or less frequent themes) and can lead to losing the participant’s perspective by creating too much distance from the data [68]. Counts of qualitative data also require more structured data collection that can hinder opportunities for unexpected or novel findings [68].

However, qualitative researchers may have appropriate reasons to enumerate. Enumerating qualitative data can explain how small samples can produce large amounts of data (e.g., words, observations) and can help to identify and differentiate patterns that may be overemphasized or overlooked. Researchers may also use ratings of qualitative data for complex, cross-case comparisons when preparing for qualitative comparative analysis or coincidence analysis, which is a technique used to evaluate factors influencing implementation of innovations [67–69, 72–74].

Editors and reviewers should avoid requiring qualitative researchers to justify their work through numerical evidence or statistical proof. At the same time, we acknowledge that quantitative elements can complement qualitative analysis when aligned with the study’s purpose. Overall, editors, reviewers, authors, and researchers should understand that numbers and statistics may or may not be appropriate in qualitative methods [67, 68]. We encourage qualitative researchers to describe their methods and choices if/when they use numbers/statistics and avoid succumbing to the pressure to quantify qualitative work to appease editors and reviewers.

#### Misperception 5: “Qualitative analysis requires interrater reliability”

Reviewers may inappropriately recommend interrater reliability (IRR), assuming it inherently adds rigor to qualitative research. IRR refers to the extent to which two or more individuals agree when independently applying codes or segmenting qualitative data [75]. In quantitative research, IRR is an important measure of rigor to ensure that codes are well-defined and reliably applied across teams and coded quantitative data. In qualitative coding, IRR requires specific procedures and structured data collection. First, units of analysis must be clearly defined (e.g., words, paragraphs, or some other unit) and identified across the dataset [76, 77]. Second, codes must be well-defined and illustrated with clear, marginal, and non-cases for individual coders to reliably apply codes to units [77–79].

Qualitative researchers have critiqued IRR because it overemphasizes the analysts’ learned ability to reliably apply codes to similar segments rather than whether the coded data appropriately identifies social phenomena [15, 77, 80]. IRR is often misused when reported in qualitative research and is only appropriate when following very specific approaches to qualitative designs and analysis, such as more deductive analytic techniques [77]. In implementation science, we often use diverse approaches to qualitative analysis, such as rapid qualitative analysis where coding is not used [37, 81]. We suggest that qualitative researchers prioritize interpretive criteria, whether inductively or deductively established, to identify significant social phenomena in qualitative data [37, 81]. Whereas statistical techniques are constrained both by the sensitivity of the coding scheme and the coders’ ability to reliably apply well-defined codes, interpretive techniques mobilize coders’ ability to converge on a shared understanding of participants’ meanings to generate a multifaceted analytic portrait of complex social processes [77, 82]. Qualitative research often leverages analysts’ longitudinal engagement with the research topic and experiences collecting various qualitative data type(s) to generate meaningful analytic findings [83]. Similarly, in implementation science, there is growing interest in using qualitative methods to understand longitudinal pathways from implementation to sustainment and whether determinants and outcomes change over time [84].

Instead of IRR, we advocate developing and implementing a rigorous, iterative process to foster interpretive analytic convergence based in qualitative methodological techniques. For example, team-based qualitative research often uses consensus methods instead of IRR [85]. Consensus-based approaches leverage multiple qualitative researchers’ experience collecting data sometimes from different geographical sites, different data types, or key constituent types to facilitate discussion about qualitative data management and analysis [79, 85, 86]. Once collected, research teams convene to discuss the data for overall observations and ideas across the data set(s). Using iterative processes, researchers propose and revise codes and preliminary ideas, and discuss how to construct and refine analyses. Qualitative researchers can use consensus as the main decision-making tool from initial to final analyses. In sum, we encourage editors and reviewers to understand that IRR is often inappropriate for qualitative research and does not inherently signify analytic rigor. Instead, qualitative researchers should describe the rigorous and iterative processes that are appropriate for the analytic approach being used.

### Misperceptions 6–10. Applying inappropriate qualitative standards

#### Misperception 6: “Qualitative research requires saturation”

Saturation has inappropriately been considered a universal standard for rigorous qualitative research [15]. The term “saturation” was first used by Glaser and Strauss (1967) in their original formulation of the grounded theory method, which they described as the point in which “no additional data are being found whereby the [researcher]. can develop properties of the category” [87]. More recently Saunders et al. [88] characterized saturation for qualitative research functionally “as a criterion for discontinuing data collection and/or analysis” (p. 1893) and describe that this criterion has achieved orthodoxy as a proxy for the overall quality of qualitative research.

Qualitative authors often fail to define what they mean by the term ‘saturation’ and where and how saturation was applied in their qualitative research process (i.e., in relation to sampling or analysis or both) [45, 89]. Further, depending on the qualitative research tradition employed, a qualitative study may not benefit from using saturation, but rather rely on other process or quality criteria concordant with the specific research tradition. For example, instead of focusing on “saturation” in implementation studies, researchers may consider whether or not their sample has high “information power” [28, 43–45, 90]. Information power indicates that the more information the sample holds, relevant for the study, the lower number of participants is needed [44]. Higher and lower information power is based on narrow/broad specific aims, dense/sparse sample, presence/absence of theory, strong/weak dialogue with participants, and case/cross case analysis [44]. Specifically, implementation researchers often design studies to yield high information power by incorporating focused aims on understanding implementation determinants, engaging key informants with highly specific knowledge, and using theory to inform study design and explain complex implementation processes and outcomes [62]. Similarly, consider whether the sample is sufficient to answer the research question, which may vary depending on scope of the inquiry and whether the work is informed by theory or seeking to develop a theory [90].

Overall, saturation is not always appropriate nor is it a universal marker of rigorous qualitative research. Other approaches such as information power or sufficiency may be more appropriate [15, 28, 43–45, 90, 91]. If saturation is used, authors should specify which type and why it is relevant for the study, and how and when it was assessed.

#### Misperception 7: “Qualitative research requires member checking”

Reviewers inappropriately expect qualitative researchers to use member checking, believing it is a “gold standard” for rigorous qualitative methods [15, 36, 92–94]. Member checking is typically characterized as inviting study participants for a post-hoc review of data or results to ensure the researcher has reflected participants’ experiences and perspectives in the reports or findings [16, 36, 93]. Member checking techniques can be conducted formally or informally and are often used to validate research processes to bolster the credibility of research findings [36, 93, 95]. However, member checking practices vary widely and have been subject to critique within qualitative research methodology [11, 15, 16, 28, 92, 94–97].

Although member checking is often used to promote rigor, qualitative methodologists have argued that when used unreflexively, member checking practices can threaten validity. Participants are often under-prepared to discern how researchers’ results are accurate, especially if findings are anonymized, aggregated, and decontextualized [11, 16, 92, 97]. Participants may ask to correct, remove, or revise raw data or analyzed results to create a new perspective [16, 28, 96, 97]. As a result, researchers may have difficulty reconciling differences between participants’ proposed changes and the researcher’s established qualitative data management and analysis procedures when interpreting the data [15, 16, 94]. Finally, member checking can add to participant burden and can cause discomfort, especially if participants revisit trauma or sensitive topics [96, 98].

When member checking is used, it requires reflexivity, defined as the process of critically examining one’s own position, assumptions, and influence throughout the research process [99]. Reflexivity involves clarifying the purpose of member checking, whether to validate findings, co-construct meaning, or invite critique. Reflexivity also guides researchers to interpret participant feedback critically by recognizing that differing perspectives may reveal multiple legitimate interpretations rather than analytic error.

We recommend thoughtful description about how qualitative researchers enact validity and verification procedures during the research process while research is underway [16, 97]. For example, interviewers should ask clarifying questions during data collection to ensure participants’ perspectives are understood and appropriately documented [16, 28, 97]. This is standard in rigorous qualitative methods and should be prioritized. In implementation research, member checking is often impractical because participants, such as busy frontline staff, often do not have time to review data or correct results. Instead, implementation researchers should encourage collaborative communication during data collection and if appropriate for study aims, could use *periodic reflections* to ask follow-up questions to understand implementation processes over time [16, 100].

Overall, we encourage reviewers and authors to carefully consider how post-hoc member checking is included in research given these methodological critiques [11, 16, 28, 92, 95–97]. Researchers should strive to incorporate iterative processes to establish validity and verification throughout the research process [16, 97]. When post-hoc member checking is included, authors should be explicit in the reasons they use this process, how it was incorporated into regulatory procedures and consent documents, and procedures should clearly describe how participant feedback was included as part of the research process.

#### Misperception 8: “Qualitative analysis requires coding”

Reviewers of qualitative research often inappropriately expect qualitative researchers to use line-by-line coding as part of their approach to data analysis because it is believed to be more systematic and replicable [13]. Coding refers to clustering and labeling pieces of data (e.g., relevant chunks of text) by topic or theme so that conceptually related data can be organized for later review and synthesis. For many years, the literature on qualitative analysis focused primarily on diverse approaches to coding as a way of organizing and distilling meaning from qualitative findings, such as grounded theory method coding, deductive coding, qualitative content analysis techniques, among many others [101–103]. Although coding is only one of many analytic tools, it has come to be taken as a reassuring and necessary signal of analytic thoroughness.

Although coding can be effective in analyzing qualitative data, it is one of many equally valid approaches increasingly called upon in qualitative research, especially in implementation science [13, 17]. Similarly useful approaches to qualitative analysis include rapid qualitative analysis [17, 37, 81], diagramming [104], matrix analysis [105], “Sort and Sift, Think and Shift” [106], and more interpretive/open-ended approaches [13, 18, 107]. Each technique offers a structured sequence for organizing data and identifying patterns of meaning relevant to the research questions. Implementation studies have compared analytic approaches used in reviewing a single dataset. For example, comparisons of traditional coding and rapid qualitative analysis found that both approaches produce similar findings when conducted by experienced teams with expertise and appropriate training [81, 108, 109].

When considering whether a proposed/described analytic approach is appropriate for the research question(s) and dataset, we encourage reviewers to look beyond the expectation of coding to consider whether the steps the qualitative researcher has undertaken for organizing and identifying meaning are clearly described, logical, and systematically and reflexively applied across the dataset or to a specified subset as appropriate [17, 110].

#### Misperception 9: “Qualitative results require themes”

Reviewers and journal editors often expect themes as part of the results section because it is a common approach to presenting qualitative data. Thematic analysis, popularly systematized by Braun & Clarke (2006) [111], is one of the most common qualitative data analysis approaches. However, not all qualitative research questions call for generating themes, in part because themes are not the only way to report or present qualitative findings.

When authors use the word “theme,” it is often undefined or under-defined, which raises questions about what authors mean by this term that has been variably defined [112]. Themes exist in literary criticism, linguistics, and social science research. In literary criticism, thematic structure [113] refers to formal patterns in a text to identify the overall “salient abstract idea” in a single text (Baldick, 2015, p. 333) [114]. Within social science, Braun and Clarke [111] systematized a six-step approach to thematic analysis and discuss themes as *the result* of the analytic process, rather than “entities that reside in data, pre-existing any analytic work on the part of the researcher” (2021, p. 39) [115].

While themes are a common type of analytic finding, they are not the only reportable finding. Each analytic approach has other, equally robust ways for characterizing social life, local contexts, and meaning. Some qualitative research reports topics, domains, or even categories derived across a dataset. For example, qualitative content analysis aims to describe the full range of phenomena in a dataset using deductive, inductive, or a combination where the result may be repeated patterns across a data set [116, 117]. In implementation science, rapid qualitative analysis is commonly used because it supports rapid turn-around of results to inform implementation [18, 37, 81, 116]. Ethnographic analysis can report social behavior using different units of analysis that range from an individual social act or action, an activity, a situation, process, or norm [118–120].

Overall, while themes are a prevalent type of qualitative result, themes are by no means the only meaningful findings. Reviewers should assess how authors define their units of analysis and what results are appropriate for the study. Authors should clearly cite prior methodological literature to justify their analytic processes and results.

#### Misperception 10: “Qualitative Data Analysis Software is necessary”

Reviewers often expect qualitative researchers to use Qualitative Data Analysis Software (QDAS). QDAS platforms (e.g., NVivo, ATLAS.ti, and MAXQDA) support facets of qualitative data management [121–123]. QDAS can structure and organize data to facilitate the researcher’s identification and development of data segments, codes, reflective or analytic memos, diagrams, and preliminary themes. QDAS also assists qualitative researchers in searching and interrogating data sources by linking parts of data together using codes, memos, and other software features. While QDAS can assist with finding linkages and patterns in data, it is not conducting analysis independent of the researcher.

QDAS is not always needed for qualitative research processes [124]. For example, rapid qualitative analysis, a widely used implementation science approach designed to produce timely feedback for sites and operational partners [37, 81], rarely employs QDAS due to the time and training required for effective use. Instead, teams typically develop summaries in Microsoft Word or analytic matrices in Microsoft Excel to create collaborative outputs accessible without specialized software expertise [81, 111]. Qualitative researchers have many ways to approach data management and analysis with rigor. QDAS facilitates aspects of data management, but like any tool, depends on how skillful the tool is used towards those analytic ends. Qualitative researchers should describe which tools they use and how they use them to manage their data and sufficiently describe their analytic questions and process.

## Discussion

Qualitative research methods are essential to implementation science, and other disciplines, to understand critical information about the why, how, and what of human experiences and social contexts [19]. Despite the centrality of qualitative methods in implementation science, those who use qualitative methods frequently encounter barriers during the dissemination process, including journal editor screening and peer-review, that may negatively impact funding and publishing opportunities. We identified and dispelled 10 misperceptions about qualitative methods and provided specific considerations for implementation science. Misperceptions 1–5 focus on reviewers’ inappropriate application of quantitative evaluation standards to qualitative methods, including perceptions that qualitative methods are too subjective, sample sizes are too small to be meaningful, qualitative results are not useful because they are not statistically generalizable, numbers and statistics are needed to legitimize results, and interrater reliability enhances coding rigor. Misperceptions 6–10 focus on reviewers’ inappropriate application of overly prescriptive qualitative standards, which involve universal expectations for saturation, member checking, coding, themes, and use of Qualitative Data Analysis Software.

To help dispel these misperceptions, we use prior literature and our expert group’s experiences to develop guidance for editors, reviewers, authors, and researchers. Our goal is to inform qualitative researchers and those who evaluate qualitative manuscripts (and proposals) to adopt a more nuanced, informed approach to writing and reviewing qualitative work [1–3, 11, 28, 33, 59]. Thus, we provide a concise, but also comprehensive resource of responses and references about key issues and common barriers that curtail the funding and dissemination of qualitative research findings for implementation science and other disciplines. Our work also helps dispel misunderstandings or misapplications of standard reporting checklists [15]. Perhaps counterintuitively, these standards can contribute to methodologically *incongruent* reporting [15]. Misuse of checklists further underscores the importance of including qualitative methodologists/investigators on research and evaluation teams, so that qualitative methods are conducted rigorously and thoughtfully. We offer recommendations for qualitative researchers, analysts, methodologists, and authors to dispel these 10 misperceptions to prepare them preemptively when writing grants, manuscripts, and ethics protocols and when engaging with other researchers and operational partners.

We also offer guidance to editors and reviewers of journal articles and grants to be aware of and help dispel these 10 common misperceptions about qualitative research. We encourage sharing our guidance with manuscript and grant reviewing teams to ensure best fit matches between prospective reviewers and the methodologies that they will be assessing. Editors and reviewers can also use the “check your misperceptions” tool to identify whether they are suitable to review qualitative methods.

### Limitations

We selected misperceptions based on salient issues from our collective experience in publishing, mentoring, and reviewing qualitative methods as well as in our role as researchers, peer-reviewers, journal editors, teachers, and mentors. However, this is not an exhaustive collection of all misperceptions and challenges that may affect qualitative research funding and dissemination efforts. We anticipate additional need for future work in the area, recognizing that misperceptions evolve over time. For example, misperceptions around the use of generative Artificial Intelligence (genAI) in qualitative research is likely an important topic that will need to be addressed in the future [125, 126].

## Conclusions

Researchers in implementation science, health services research, and clinical trials need qualitative research methods to understand human behaviors and local contexts that affect health and implementation outcomes [19, 70]. Qualitative research provides insight into human behavior, situated local contexts, and social processes in ways that cannot be understood using solely quantitative methods. Qualitative methods provide researchers and program evaluators with rich information to understand if and how evidence-based innovations are or should be implemented, sustained, and adapted to better meet the needs patients and providers [19, 71, 84]. Although interest in qualitative and mixed methods continues to expand, qualitative methods have historically been more difficult to fund and publish due to longstanding cultural beliefs and values that favor quantitative methods.

Thus, we developed guidance to dispel general misperceptions about qualitative methods while also addressing specific considerations for implementation research that can be applied across diverse study designs, implementation frameworks, and qualitative theoretical traditions. Journal editors and reviewers can use our guidance to avoid inappropriate critiques and provide more thoughtful and appropriate evaluations of qualitative research and decisions about funding and publishing. Further, this manuscript is a resource for authors to strengthen their writing by anticipating and preventing common critiques of qualitative research. By describing 10 key misperceptions, we emphasize the importance of more impartial and appropriate qualitative methods review, so that meaningful proposals can be funded, manuscripts can be accepted, and results can be disseminated, which is critically important to implementation science and other disciplines.

Our recommendation is for reviewers and editors to be mindful of how qualitative methods standards differ from quantitative methods standards rather than enforce inappropriate standards. We also urge journals editors to be more selective in whom they choose to review qualitative work. It is also an ethical obligation for reviewers with quantitative preferences and biases to understand their limitations in reviewing qualitative methods and recuse themselves from reviewing qualitative manuscripts when appropriate. Overall, we hope editors and reviewers will be more mindful of how their ideologies may impact their perceptions and reviews of qualitative methods and question if they are well-suited to review qualitative work, so that important qualitative research is not overlooked or discounted.

## Supplementary Information

Additional file 1. Questions to check misperceptions about qualitative methods.

## Data Availability

Not applicable.

## Abbreviations

QualRIS: Qualitative Research in Implementation Science
TMFs: Theories, Models, and Frameworks
CFIR: The Consolidated Framework for Implementation Research
RE-AIM: Reach, Effectiveness, Adoption, Implementation, and Maintenance framework
IRR: Interrater reliability
QDAS: Qualitative Data Analysis Software
