# Supplementary material for: Optimizing qualitative methods in implementation research: a resource for editors, reviewers, authors, and researchers to dispel ten common misperceptions about qualitative research methods
Source: Implement Sci. 2025 Dec 4;21:4. doi: 10.1186/s13012-025-01474-z (PMC12797730; doi:10.1186/s13012-025-01474-z)
Supplement: Supplementary file 1 — Additional file 1. Questions to check misperceptions about qualitative methods. [file 13012_2025_1474_MOESM1_ESM.docx]

## Supplemental Files

Additional File 1. Questions to check misperceptions about qualitative methods.

Instructions: Editors and reviewers can take this quiz to assess their understanding of qualitative methods and identify possible misperceptions related to qualitative methods. This quiz should be taken before reading the article and may help editors and reviewers assess their ability to evaluate qualitative methods.

| Question | True or False  (circle response) |
| --- | --- |
| 1. Qualitative methods are too subjective to provide meaningful contributions to the field. | True / False |
| 1. Qualitative sample sizes are too small to be meaningful. | True / False |
| 1. Qualitative results have limited utility because they are not statistically generalizable. | True / False |
| 1. Qualitative results need numbers/statistics to be impactful. | True / False |
| 1. Interrater reliability is required for rigorous qualitative analysis. | True / False |
| 1. Saturation is always needed in qualitative research. | True / False |
| 1. Member checking procedures are needed for all qualitative research. | True / False |
| 1. Coding is essential for all qualitative analysis. | True / False |
| 1. Qualitative analysis should always result in themes. | True / False |
| 1. Qualitative Data Analysis Software is needed for rigorous qualitative analysis. | True / False |

**Answers**: Answering true to any of the questions will identify a misperception about qualitative methods. A higher number of questions answered “True” indicates a higher level of misunderstandings about qualitative methods, which may also indicate poor fit between the reviewer and the qualitative methods being assessed.
